# Outbreak of KPC-producing *Klebsiella pneumoniae* at a Portuguese university hospital: Epidemiological characterization and containment measures

**DOI:** 10.1097/j.pbj.0000000000000186

**Published:** 2022-12-01

**Authors:** David Peres, Paulo Figueiredo, Paulo Andrade, Nuno Rocha-Pereira, Cláudia Carvalho, Rita Ferraz, Raquel Duro, Arnaldo Dias, Abel Gomes, Cláudia Pereira, Gisélia Braga, Virginia Pereira, Lino Azevedo, Edgar Moniz, Manuela Ribeiro, Eugénia Ferreira, Vera Manageiro, José Teixeira, Tiago Guimarães, Manuela Caniça, Carlos Alves

**Affiliations:** a Infection and Antimicrobial Resistance Prevention and Control Unit, Matosinhos Local Health Unit, Matosinhos, Portugal; b Infection and Antimicrobial Resistance Prevention and Control Unit, Centro Hospitalar Universitário São João, Porto, Portugal; c Infectious Diseases Department, Centro Hospitalar Universitário São João, Porto, Portugal; d Vascular Surgery Department, Centro Hospitalar Universitário São João, Porto, Portugal; e Microbiology Laboratory, Centro Hospitalar Universitário São João, Porto, Portugal; f National Reference Laboratory of Antibiotic Resistances and Healthcare Associated Infections, National Institute of Health Doutor Ricardo Jorge, Lisbon, Portugal

**Keywords:** active surveillance cultures, antimicrobial resistance, carbapenemase-producing *Enterobacteriaceae*, contact precautions, KPC-producing *K pneumoniae*, outbreak

## Abstract

**Background:**

KPC-producing *K pneumoniae* (KPC-Kp) is a public health problem with important clinical and epidemiological implications. We describe an outbreak of KPC-Kp at vascular surgery and neurosurgery wards in a central hospital in Porto, Portugal.

**Methods:**

A case of KPC-Kp was considered to be a patient positive for KPC-Kp with strong epidemiological plausibility of having acquired this microorganism in the affected wards and/or with genetic relationship ≥92% between KPC-Kp isolates. Active surveillance cultures (ASCs) and real-time polymerase chain reaction were used for the detection of carbapenemase genes through rectal swab in a selected population. Molecular analysis was performed using pulsed-field gel electrophoresis at the National Reference Laboratory. Patient risk factors were collected from the electronic medical record system. Information regarding outbreak containment strategy was collected from the Infection Control Unit records.

**Results:**

Of the 16 cases, 11 (69%) were identified through active screening, representing 1.4% of the total 766 ASCs collected. The most frequent risk factors identified were previous admission (63%), antibiotic exposure in the past 6 months (50%), and immunodepression (44%). The length of stay until KPC-Kp detection was high (0–121 days, mean 35.6), as was the total length of stay (5–173 days, mean 56.6). Three patients (19%) were infected by KPC-Kp, 2 of whom died. One previously colonized patient died later because of KPC-Kp infection.

**Conclusions:**

Multifactorial strategy based on contact precautions (with patient and healthcare professional cohorts) and ASC, as well as Antibiotic Stewardship Program reinforcement, allowed to contain this KPC-Kp outbreak.

## Background

Multidrug resistance has been growing worldwide, representing a big concern to the scientific and medical community. Carbapenemase-producing *Enterobacterales* (CPE), such as *K pneumoniae*, are a global public health problem nowadays from the epidemiological, microbiological, clinical, and infection control perspectives.^1–5^ The transmission of CPE can occur in both healthcare and community settings and even across borders.^6,7^ Hospitalized patients are more susceptible to infections, increasing the risk of morbidity, mortality, length of stay (LOS), and healthcare costs.^8–10^ Many outbreaks by CPE have been reported worldwide,^11–15^ including European countries (Finland,^16^ Germany,^17^ Switzerland,^18^ France,^19^ Spain,^20^ Greece,^21^ and Italy^22^). In response to this problem, several articles with interventional strategies and guidelines have been published.^8,23–26^

Carbapenem-resistant *K pneumoniae* in Portugal has been monitored, and since 2008, there is an increase in the number of isolates from blood and cerebral spinal fluid (from <1% in 2008, 1.8% in 2013, 3.4% in 2015 to 5.2% in 2016).^27^ A study by Manageiro et al, including 2105 *Enterobacteriaceae* collected between April 2006 and February 2013, described the molecular epidemiology of CPE in Portugal and the predominance of the carbapenemase KPC-3. In this article, 165 isolates nonsusceptible to carbapenemes were reported, from which 35 were carbapenemase producers (distributed in all years since 2010 to 2013 and collected from different samples, mostly urine) and were identified in 5 different bacterial species, of which 22 were *K pneumoniae.*^28^ According to the 2015 epidemiological analysis from the European Center for Disease Prevention and Control (ECDC), Portugal is at an intermediate epidemiological stage for CPE.^29^ However, the prospect of its growth should inspire concern, considering the extensive resistance profile and apparent ease to generate outbreaks.^27^ In this article, we describe an outbreak of KPC-producing *K pneumoniae* (KPC-Kp) isolates, and the containment measures adopted, that occurred in 2016 in the vascular surgery and neurosurgery wards of a university hospital center in the north of Portugal.

## Methods

### Setting

Centro Hospitalar Universitário São João (CHUSJ) is a university hospital center constituted by 2 units: the main one with 1056 beds and the secondary one with 49 beds. Situated in Porto, with a city of 238,000 inhabitants in Portugal, it is the biggest hospital in the north of Portugal, with 5600 professionals. In 2016, 44,972 inpatients were treated. The outbreak of KPC-Kp occurred from January to July 2016. Two wards were affected: the vascular surgery ward (44 beds) and neurosurgery ward (32 beds), both located in the main unit. Before the outbreak, in 2015, CHUSJ had 548 cases of ESBL-producing *Enterobacteriaceae* (1.57 cases per 1000 patient-days) and 74 cases of carbapenem-resistant *Enterobacteriaceae* (0.21 cases per 1000 patient-days), most of which were noncarbapenemase producers.

### Definitions

 Active surveillance culture (ASC): screening for the presence of KPC-Kp with the aim to implement infection control measures to avoid cross-transmission.

Outbreak case of KPC-Kp: patients positive for KPC-Kp with strong epidemiological plausibility of having acquired this microorganism in the affected wards and/or with genetic relationship ≥92% between KPC-Kp isolates between January and July 2016.

Contact precautions: use of gown and gloves in the contact with patient or its close environment.

Immunodepression: presence of any condition or treatment known to produce immunodepression, such as malignancy, cirrhosis, or use of immunosuppressors.

Risk factors for KPC-Kp: based on the available literature,^1,25,30^ the following risk factors were considered: diabetes mellitus, organ transplant, dialysis, prosthesis, skin ulcers, chronic urinary catheter, nasogastric tube, dependency to body hygiene self-care, immunodepression, neoplasm, antibiotic exposure (previous 6 months), surgery (previous 6 months), hospital admission (previous 6 months), and other past positive results for multidrug-resistant microorganisms (MDROs) (previous 12 months).

### Bacterial identification and antibiotic susceptibility

Microorganism identification was performed using suitable culture media according to the protocols established in the microbiology laboratory. Genus and species were identified using the MALDI-TOF methodology (Vitek MS from bio Merieux, Lyon, France) and the presence of carbapenem resistance was analyzed by antimicrobial susceptibilities in the bioMerieux Vitek2 automatic equipment, complemented with manual methods of disk diffusion (Kirby-Bauer) and gradient concentration (E-test, bioMerieux, Lyon, France), according to the European Committee on Antimicrobial Susceptibility Testing (EUCAST) guidelines.

### Molecular epidemiology

The presence of carbapenemase was confirmed using a real-time polymerase chain reaction (RT-PCR) method, GeneXpert (Cepheid, Sunnyvale, CA, EUA), which allows identification of KPC-encoding, NDM-encoding, VIM-encoding, OXA-48-encoding, and IMP-encoding genes.

ASCs were performed using rectal samples collected by dry swabs and swabs with transport medium. Dry swabs were used to investigate the presence of the carbapenemase gene using the RT-PCR method, GeneXpert (Cepheid). The swabs with transport medium were used, in PCR positive cases, to perform the cultural examination for microorganism identification, as mentioned above.

The carbapenemase-producing strains were subjected to pulsed-field gel electrophoresis (PFGE) at the National Institute of Health Doutor Ricardo Jorge, Lisbon, Portugal, as previously described.^31^

### Infection control

This health unit has an Infection and Antimicrobial Resistance Prevention and Control Unit, integrated in the Hospital Epidemiology Center, which performs its activity in 5 vital areas: epidemiological surveillance, practice standards, training, auditing and consultancy, and antimicrobial stewardship. There is a local policy of standard precautions (in accordance with the ongoing national campaign) and additional precautions. Every patient colonized or infected with MDRO is cared according to contact precautions; in case of CPE, patients are housed either in a single room or in a cohort.

From 2015 to 2018, CHUSJ was one of the 12 hospital centers involved in a national project to reduce the 4 main healthcare-associated infections (surgical site, ventilator-associated pneumonia, central venous catheter (CVC)–related bacteremia, and urinary catheter–associated infection) through the implementation of infection prevention bundles.

### Ethics

This report was approved by the Ethics Committee for Health of Centro Hospitalar Universitário São João (Reference No. 190-17).

## Results

**Figure 1.**
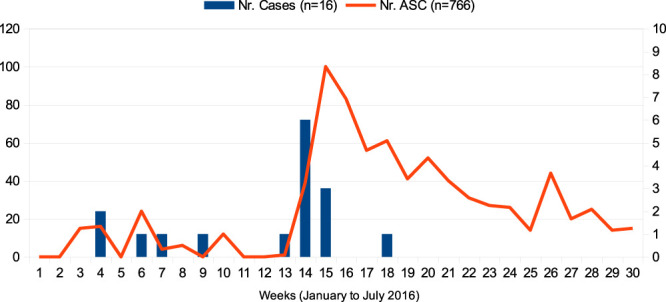
Time evolution of cases (considering sample collection date) and ASC collected during the KPC-Kp outbreak—São João Hospital Center, Portugal, 2016. ASC, active surveillance culture (rectal swab); KPC-Kp, KPC-producing *Klebsiella pneumoniae*.

On April 7, 2016 (week 14 in Fig. 1), an alert from the microbiology laboratory indicated a carbapenem-resistant KPC-Kp that has been detected at the tip of a CVC removed from a vascular surgery inpatient. A 74-year-old man has been admitted in December 2015 with an endovascular prosthesis infection (patient 6, Table 1). Contact precautions were implemented immediately, and ASCs were collected from contacts, which gave rise to identification of other cases (patients 7–12). An outbreak was declared, and infection control measures were implemented, with the closure of the ward to new admissions and stricter epidemiological surveillance.

**Table 1:** Characteristics of cases of KPC-Kp Outbreak (n = 16); Centro Hospitalar Universitário São João, Portugal, 2016 (sorted by chronological order of agent identification).

Ref.	Sex, age (y)	Diagnosis	Ward	Number of risk factors*	LOS to KPC (d)	Total LOS (d)	Type of sample	PFGE clone	Infection/colonization	Immediate outcome	Follow-up after 1 year	Death due to KPC-Kp
1	M, 84	Bone fractures (multiple myeloma)	Orthopedics	3	31	33	Sputum	001	Infection	Died	—	Yes
2	M, 52	Brain tumor	Neurosurgery	5	4	43	Urine	001	Colonization	Other hospital	Died	No
3	F, 84	Congestive heart failure decompensation	Medicine	7	5	24	Urine	001	Colonization	Died	—	No
4	M, 81	Aspiration pneumonia	Orthopedics/medicine	11	0	7	Sputum	001	Colonization	LTCF	Died	No
5	F, 81	Renal decompensation	Medicine	5	4	5	Urine	001	Colonization	Home	Died	No
6†	M, 74	Peripheral arterial disease	Vascular surgery	4	97	138	Rectal swab/CVC	001	Infection	Died	—	Yes
7	M, 55	Peripheral arterial disease	Vascular surgery	0	26	52	Rectal swab	No info‡	Colonization	Home	Alive	—
8	M, 69	Peripheral arterial disease	Vascular surgery	1	19	44	Rectal swab	No info‡	Colonization	LTCF	Alive 2 ASC (−)	—
9	M, 80	Peripheral arterial disease-infected toes	Vascular surgery	3	52	79	Rectal swab/Wound	001	Infection	Home	Died	Dubious
10	M, 84	Prosthesis infection	Vascular surgery	4	12	14	Rectal swab/Wound	001	Colonization	Home	Alive	—
11	M, 64	Peripheral arterial disease	Vascular surgery	6	36	39	Rectal swab	001	Colonization (while inpatient)	Home	Died 2 ASC (+)	Yes
12	M, 72	Lower limb infected ulcers	Vascular surgery	3	8	173	Rectal swab	001	Colonization	Home	Alive	No
13	M, 59	Brain abscess	Neurosurgery/Vascular surgery	1	50	55	Rectal swab	No info‡	Colonization	Home	Died 1 ASC (−)	No
14	M, 67	Healthcare-associated meningitis	Neurosurgery	3	42	47	Rectal swab	No info‡	Colonization	Home	Died 2 ASC (−)	No
15	M, 84	Subdural hemorrhage (traumatic brain injury)	Neurosurgery	5	63	67	Rectal swab	No info‡	Colonization	LTCF	Alive	—
16	M, 66	Peripheral arterial disease	Vascular surgery	4	121	87	Rectal swab	No info‡	Colonization	Home	Alive 2 ASC (−)	—

ASC, active surveillance culture (rectal swab); CVC, central venous catheter; F, female; KPC-Kp, KPC-producing Klebsiella pneumoniae; LOS, length of stay; LTCF, long- term care facility; M, male; PFGE, pulsed-field gel electrophoresis.

* List of risk factors in Table 2.

† Index case.

‡ Impossibility to send these samples to molecular analysis because of logistic reasons.

From the 18 patients identified with KPC-Kp, molecular analysis by PFGE showed that 10 isolates had the same clonal origin (being genetically identical and clustering into the major group at a genetic distance of 0%), 2 belonged to one other clone being 100% genetically undistinguishable from one another (but genetically unrelated to the previous 10), and 6 revealed unique genotypes and were not considered to be related with the outbreak (additional file 1, Supplemental Digital Content, http://links.lww.com/PBJ/A12). Patient characteristics are presented in Table 1. Of the 16 patients who met the outbreak case definition (of which 6 did not have identification by molecular analysis), only 2 were women, age between 52 and 84 years (mean, 71.6 years), and 6 (38%) were admitted due to peripheral arterial disease. Of the 14 KPC-Kp risk factors considered, 4 were on average present (one patient had none and one had 11). The most frequent risk factors present were previous hospital admission (n = 10; 63%), antibiotic exposure in the previous 6 months (n = 8; 50%), and immunodepression (n = 7; 44%) (Table 2).

**Table 2:** Epidemiological indicators and risk factors of cases of KPC-Kp outbreak (n = 16); Centro Hospitalar Universitário São João, Portugal, 2016.

Epidemiological indicators	n (%)
Related to ASC	
Number of ASC collected (January to July 2016)	766
ASC positive result	11 (1.4)
Related to KPC-Kp cases	
Colonized cases	13 (81)
Alive as immediate outcome	13 (81)
Alive after 1 year	6 (38)
Attributable mortality due to KPC-Kp infection	3* (75)
Risk factors of KPC-Kp cases	
Diabetes mellitus	5 (31)
Organ transplant	1 (6)
Dialysis	2 (13)
Prosthesis	5 (31)
Skin ulcers	3 (19)
Chronic urinary catheter	1 (6)
Nasogastric tube	4 (25)
Dependency to autohygiene	6 (38)
Immunodepression	7 (44)
Neoplasm	6 (38)
Antibiotic exposure (previous 6 months)	8 (50)
Surgery (previous 6 months)	5 (31)
Hospital admission (previous 6 months)	10 (63)
Previous positive result for MDRO (previous 12 months)	2 (13)

ASC, active surveillance culture; MDRO, multidrug resistant organism; KPC-Kp, KPC-producing Klebsiella pneumoniae.

* Data related to confirmed infections by KPC-Kp (includes follow-up period).

The LOS until the detection of KPC-Kp ranged from 0 to 121 days (mean, 35.6 days) and total LOS from 5 to 173 days (mean, 56.6 days). Most of the cases were detected through ASCs (n = 11; 69%), but this MDRO was also identified in 3 specimens of urine, 2 sputum, 2 wounds, and one at the tip of a CVC (Table 1).

Eighty percent of the patients with KPC-Kp involved in the outbreak (n = 13) were alive at discharge (Table 2)and 38% (n = 6) after 1 year There were 3 deaths considered to be KPC-Kp associated (and one in which this relation was dubious). Of the 766 ASCs collected during the outbreak, 11 were positive (1.4%).

## Discussion

### Outbreak detection and patient risk factors

The index case (patient 6) had several infectious complications and, 138 days after admission, died because of an infection of his right amputation stump, where KPC-Kp was isolated. The patient had, since 2007, a history of several surgeries and infectious events related to his pathology. The risk factors identified were previous hospital admission, surgery, and exposure to antibiotics in the past 6 months. In fact, a recent case–control study demonstrated that patients with prolonged hospital exposure (>28 days) are more likely to acquire KPC-Kp, suggesting that risk factors for KPC-Kp acquisition may be shared with those for vancomycin-resistant *Enterococcus.*^32^

Regarding LOS, its mean was 56.6 days and the time until the KPC-Kp detection was 35.6 days. As stressed by ECDC, there is a risk of KPC-Kp dissemination in patient transfer between healthcare facilities, with special emphasis on cross-border transfer.^33^ To the best of our knowledge, none of the cases had been traveling abroad. Patients who were transferred to other health units or discharged to long-term care facilities (LTCFs) were flagged to avoid further cross-dissemination in the units that received them. Recently, worrying findings from a Brazilian study indicated that 18% of patients were colonized at admission of emergency department with carbapenem-resistant *Enterobacteriaceae*, of whom 6 patients had no previous contact with health care, suggesting transmission of this MDRO within the community.^34^

**Figure 2.**
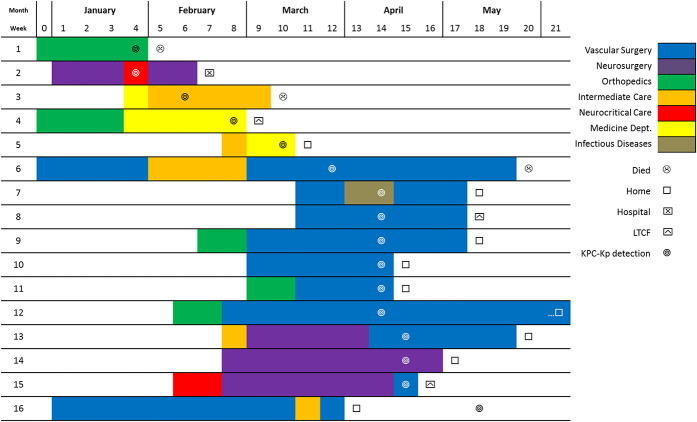
Spatiotemporal description of KPC-Kp outbreak cases (n=16)—São João Hospital Center, Portugal, 2016. KPC-Kp, KPC-producing *Klebsiella pneumoniae*; LTCF, long-term care facility.

Considering patient 6 (the index case) and by the interpretation shown in Figure 2, we could formulate the hypothesis of possible cross-transmission between patient 6 and patients 3 and 5 at the Intermediate Care Unit and between patients 1 and 4 at the orthopedics ward. Patients 6 to 16 (excluding 14) had in common the admission to the vascular surgery ward and 13 to 15 to the neurosurgery ward. Through patient record review, the possibility of cross-transmission at the imaging unit was investigated, but no common factors were found. The relationship of patient 2 to this outbreak is less clear, but the PFGE analysis confirms that it belongs to the same clone (Table 1). However, patient 2 had risk factors for KPC-Kp acquisition: hospital admission and surgery in the previous 6 months and the diagnosis of cancer. After 43 days, he was transferred to another hospital where he received palliative care until he died 2 months later.

A surveillance strategy was implemented (with the construction of an epidemiological timeline) and the first ASC official protocol was approved on April 12 (week 15 in Fig. 1), stipulating, as high-risk population, all patients admitted in the Vascular Surgery and Neurosurgery wards in the previous 6 months. This protocol was reviewed on April 16 to include, as high-risk for KPC-Kp, all patients transferred from other health units. As a result of this strategy, more cases were found, namely patients 13 to 16 (other ASCs were positive but did not meet the outbreak case definition, namely epidemiological plausibility of having acquired this microorganism in the affected wards) (Fig. 1).

### Infection control measures

When the outbreak was declared, stricter infection control procedures were put in place to address it (Table 3), namely reinforcement of standard precautions (more alcohol-based hand solutions and exclusive noncritical equipment, such as sphygmomanometers and stethoscopes, were made available); contact precautions in patients colonized or infected with KPC-Kp (with reinforcement of warning signaling and flagging in the patient medical record); implementation of the ASC protocol (as mentioned previously); and patient and healthcare worker (HCW) cohorts divided into 3 risk areas (high risk—patients who are tested positive for KPC-Kp; medium risk—patients with risk factors, waiting for ASC results; and low risk—KPC-Kp–negative). Meanwhile, another ward that had just been renovated (and as yet unoccupied) received the new admissions for vascular surgery after ASCs (low-risk area). It was also implemented continuous surveillance (through an epidemiological line list); frequent briefings (with hospital administration, medical, and nursing coordinators of the affected wards, epidemiology center, and microbiology and pharmacy professionals); relocation of the eco Doppler examination room (which was originally in the high-risk area); daily bathing of KPC-Kp cases with chlorhexidine 4%; frequent training sessions for HCWs on contact precautions procedures and daily auditing of practices with immediate feedback; restriction of visitors and medical students; improvement of intrahospital information and patient flow (definition of inpatient circuits, with emphasis on hemodialysis, angiography, operating room, postanesthetic unit, and outpatient clinic); and interhospital information (patient flagging before discharge to outpatient units or transfer to other health units or LTCFs). The effectiveness of environment cleaning was guaranteed by the daily presence and supervision of an infection control nurse at ward procedures and HCW's shift changes. There was also enforcement and supervision of the equipment decontamination protocol (daily cleaning with detergent and sodium hypochlorite 1% and terminal decontamination with hydrogen peroxide technology). In fact, in a recent review reporting containment measures of 13 KPC-Kp outbreaks, 9 (69.2%) applied enhanced environmental cleaning.^11^ The Antibiotic Stewardship Program (ASP) was also reinforced, namely revision and implementation of surgical prophylaxis protocol, policy implementation of diabetic foot infection treatment (identified as the major cause of antibiotic usage in the affected area), and reinforcement of good practices related to collection of biological products and implementation of antibiotic stewardship interventions (audit and feedback to prescribers, restricted use of carbapenems).

**Table 3:** Description of KPC-Kp outbreak containment measures; Centro Hospitalar Universitário São João, Portugal, 2016.

IC measures at KPC-Kp outbreak
1. Reinforcement of standard precautions 2. Contact precautions in patients colonized or infected with KPC-Kp 3. Patient and HCW cohorts (divided in 3 distinct risk areas) 4. Implementation of ASC protocol 5. Continuous surveillance through epidemiological line list 6. Daily bathing of KPC-Kp cases with chlorhexidine 4% 7. Daily presence of infection control nurse at ward procedures and HCW's shift changes 8. Environmental and equipment decontamination protocol supervision 9. Relocation of eco Doppler examination room 10. HCW's training sessions on contact precautions procedures 11. Visitors and medical students mobility restriction 12. Intrahospital information and patient flow (definition of inpatient/outpatient circuits) 13. Interhospital information (patient flagging before discharge or transfer) 14. Antibiotic Stewardship Program reinforcement

ASC, active surveillance culture (rectal swab); HCWs, healthcare workers; KPC-Kp, KPC producing *Klebsiella pneumoniae*.

The infection control measures implemented were based on the published guidelines.^23–26^ The 2 central cornerstones (contact precautions and ASCs) are considered by the European Society of Clinical Microbiology and Infectious Diseases guidelines as a “moderate evidence” but “strong recommendation.”^26^ On the other hand, measures such as patient bathing with chlorhexidine and antibiotic stewardship are considered in these guidelines as “low/very low evidence” and “conditional recommendation.” Considering this was an outbreak setting, it is not possible to extrapolate the individual effect of the infection control measures because they were implemented simultaneously. In fact, in a recent review, French et al^8^ found that CPE outbreaks can be controlled using a combination of measures, but the quality of the evidence is still weak, especially on the effectiveness of individual infection control measures.

Since this outbreak, a policy to prevent and control KPC-Kp at this Hospital Center is in place. This includes risk evaluation at admission, namely all patients coming from other health units or LTCFs (with admission for more than 48 hours) and flagged at patient medical records (as direct contacts of cases) and those with previous admission to the affected wards. If any of these risk factors is present, an ASC is collected and contact precautions are instituted until the result is available. As stated by Vubil et al,^35^ implementation of carbapenemase gene screening is advisable on hospital patient admission to control KPC-Kp dissemination. Our hospital protocol also includes decontamination instructions for equipment and environment, guidance on how to collect ASCs, written information for patients and caregivers, and guidelines for outpatients with KPC-Kp (which have different circuits from other patients). A specific recommendation is available to discontinue special measures of patients with KPC-Kp, namely: more than 12 months since the last positive result and no antibiotic use in the past 30 days and 3 negative RT-PCR rectal swabs (with at least a 48-hour interval between each other) and absence of other possible source of colonization or infection (such as ulcers or chronic urinary catheter). These 4 criteria should all be fulfilled to remove the KPC-Kp flagging from the patient medical record. In fact, as reinforced by Asensio et al, it is important to have a dedicated plan to prevent the spread of CPE at the hospital level. This plan should be mainly supported by the management of patients (at admission and new cases during hospital stay), ASC and definition of high-risk groups.^24^

### Epidemiological indicators and patient outcome

Two patients with infection due to KPC-Kp (2/16, 12.5%) died in the hospital. At the 1-year follow-up, only 6 patients were alive (6/16, 37.5%), but after reviewing the cases, only one death was clearly KPC-Kp–related (patient 11), and in one (patient 9), this relationship was dubious. This mortality could be explained by the presence of various comorbidities in this population, representing an overall low functional status. Regarding patient 11, he was identified as colonized by KPC-Kp 36 days after being admitted and was discharged 3 days after. During his admission, he went through 3 surgeries, of which 2 were amputations of lower limb due of severe ischemia. One month later, he was readmitted due to infected necrosis of his amputation stump, where KPC-Kp was identified. After 107 days, he died because of this infection. Schechner et al.^36^ studied who were prone to become clinically infected in a population colonized with CPE and found the following independent predictors: admission to the intensive care unit, having a CVC, receipt of antibiotics and diabetes mellitus. The 2 latter risk factors were present in patient 11.

Based on 13 KPC-Kp outbreak reports, Campos et al^11^ found that the risk of becoming infected was 52% ± 20.8%. These results contrast with our findings that indicate a risk of 12.5%. The same authors described an attributable mortality rate due to infection between 27.8% and 66.7%, while Akova et al^25^ reported this rate to be between 18.9% and 48.0%. If we consider only the population with confirmed infection due to KPC-Kp and include the follow-up period, our result is higher (3/4; 75.0%).

## Conclusions

KPC-Kp outbreaks are increasingly more common and a cause of public health concern. In this report, we describe a KPC-Kp outbreak in a Portuguese university hospital center and the multifactorial strategy implemented to control it, which included reinforcement of standard precautions, implementation and supervision of contact precautions (with patients and HCWs cohorts, 3 risk areas, and specific patient circuits), an active screening protocol, and increment of the ASP. Continuous epidemiological surveillance, HCW's training sessions, environment and equipment decontamination supervision, and improvement of intrahospital and interhospital communication were also relevant to contain this outbreak.
